# Linkage of Routinely-Collected Maternity Data with a Cerebral Palsy Register: Comparison of baseline maternal characteristics in offspring with and without cerebral palsy.

**DOI:** 10.23889/ijpds.v7i3.1907

**Published:** 2022-08-25

**Authors:** Lisa Kent, Claire Kerr, Karen McConnell, Oliver Perra, Kelly-Ann Eastwood

**Affiliations:** 1 Centre for Public Health, Queen’s University Belfast; 2 Northern Ireland Cerebral Palsy Register, School of Nursing and Midwifery, Queen’s University Belfast; 3 School of Health Sciences, Ulster University; 4 Queen’s University Belfast; 5 Fetal Medicine, University Hospitals Bristol & Weston NHS Foundation Trust

## Objectives

This feasibility and hypothesis generating study aimed to compare maternal characteristics between pregnancies in which at least one infant received a diagnosis of CP (case pregnancies) and pregnancies in which all infants did not receive a diagnosis (control pregnancies).

## Approach

Data for individuals with cerebral palsy (CP) born between 1990 (commencement of Northern Ireland Maternity System (NIMATS)) and 2012 (latest fully validated birth year in Northern Ireland Cerebral Palsy Register (NICPR)) were transferred to the Honest Broker Service, who performed linkage to NIMATS. Offspring were primarily matched between NICPR and NIMATS using the unique Health and Care Number. Where this was unavailable in NIMATS, matching from offspring to mother-infant pairs was performed using surname, gender, date of birth and/or postcode. Chi-square tests were used to test for differences in maternal characteristics between case and control pregnancies.

## Results

Cohorts consisted of 486 case and 266,260 control pregnancies. After missing values were removed, case pregnancies had a greater proportion of mothers under 25 years (case: 28.0% vs control: 23.1%, p<0.05), multiple births (7.8% vs 1.5%, p<0.001), nulliparous mothers (47.5% vs 41.1%, p<0.05), unplanned pregnancies (42.8% vs 34.4%, p<0.001), late booking appointments (14.0% vs 9.7%, p<0.01), hypertension during pregnancy (11.9% vs 8.5%, p<0.05), no recorded folic acid intake (46.7% vs 34.8% p<0.001), family history of congenital abnormality (33.3% vs 24.4%, p<0.001), medical problems with potential to affect labour (respiratory, cardiac, diabetes, other) (6.6% vs 3.9%, p=0.004). A non-significant trend was observed suggesting that case pregnancies had a higher proportion of mothers with BMI≥30kg/m2 (21.4% vs 13.2%, p=0.086).

## Conclusion

This feasibility study has highlighted a range of potential maternal characteristics that may be associated with a diagnosis of cerebral palsy in offspring and mirrors findings from other studies. A future data extraction update and predictive model development is planned.

